# Innovative design and evaluation of medical nebulizer for preschool children: A user demand-driven approach

**DOI:** 10.1371/journal.pone.0325199

**Published:** 2025-12-01

**Authors:** YanXiao Zhao, Tao Wang, LiMing Zhang, Basyarah Hamat, Leah Ling Li Pang

**Affiliations:** 1 Faculty of Artificial Intelligence, Universiti Teknologi Malaysia, Kuala Lumpur, Malaysia; 2 School of Mechanical Engineering, Anyang Institute of Technology, Anyang, Henan, China,; 3 GongQing Institute of Science and Technology, Gongqing, Jiangxi, China; Jinan University, CHINA

## Abstract

**Aims:**

To enhance the user experience and satisfaction of children’s medical nebulizers, and to improve adherence and the efficacy of nebulization treatments for children, this study explores an innovative design and evaluation method for children’s medical nebulizers from the perspective of user needs.

**Methods:**

Firstly, this study conducts a thorough analysis of user behaviors and their potential needs for children’s medical nebulizers through field observations, User Journey Mapping (UJM), relevant user interviews, and the KJ method, thereby constructing a hierarchical model of demand indicators. Next, the Analytic Hierarchy Process (AHP) is employed to calculate the weight and priority ranking of each indicator, effectively identifying the crucial demand indicators that influence nebulizer design. Based on the findings from the demand analysis, an innovative design practice for children’s medical nebulizers is carried out. Finally, the fuzzy comprehensive evaluation (FCE) method is used to assess the user satisfaction of the proposed design scheme and an existing nebulizer product case used in children’s hospitals and compare the results to verify the feasibility and effectiveness of the design approach and scheme in this study.

**Conclusion:**

The results indicate that the combined application of UJM, the KJ method, AHP, and FCE can help designers more accurately capture diverse user needs, enhance the scientific rigor and rationality of design and evaluation processes for children’s medical nebulizers, and ultimately produce products with higher user satisfaction. This study contributes to the field by providing a systematic framework for the design and evaluation of preschool-aged children’s medical nebulizers, offering theoretical guidance and practical reference for future designers.

## Introduction

With worsening air pollution, the incidence of respiratory diseases in children has significantly increased, making it a leading cause of pediatric medical visits in recent years [1]. Due to its non-invasive nature, rapid efficacy, and ease of use, nebulized inhalation therapy has become the primary treatment for pediatric respiratory conditions [2]. Medical nebulizers work by atomizing medication into fine particles, delivering high-concentration aerosolized drugs directly to the respiratory tract through the mouth and nose. This enhances therapeutic effects while avoiding the potential side effects of systemic medication. However, current medical nebulizers are generally designed for adults, without fully considering the physiological and psychological particularities of children. As a result, many preschool children experience anxiety, fear, and resistance during nebulization, leading to poor treatment adherence and reduced therapeutic effectiveness [3]. This not only compromises treatment outcomes but also increases medical costs. Thus, designing specialized pediatric nebulizers is essential to improve children’s treatment experiences, alleviate negative psychological effects, and enhance adherence to therapy [4].

Existing research on medical nebulizers mainly focuses on innovation and improvement of functions and technologies, such as improving nebulization performance [5] and efficiency [6], reducing drug residue [7], and optimizing nebulization particle size [8]. However, most of these studies focus on the physical performance and technical specifications of nebulizers, with limited research on how nebulizer design aligns with the needs of specific user groups, especially children. Children have their own particularities in physiology, psychology, and behavior, which put higher demands on the design of nebulizers, such as the need to consider children’s tolerance, ease of operation, comfort during treatment, and interactive fun. Additionally, in the existing literature, there is a gap in the research on the systematic design and evaluation methods of children’s medical nebulizers. Therefore, how to effectively design children’s medical nebulizers that meet diverse user needs and establish scientifically sound evaluation methods is one of the issues that need to be solved urgently. Such research not only helps fill the current research gap in the field of children’s medical device design but also provides a scientific basis and theoretical support for future product development practices.

Effective acquisition of user demands is an essential foundation for product design and development [9]. User needs are often complex and variable, with a certain degree of obscurity, fragmentation, and ambiguity [10]. Due to children’s limited cognitive and expressive abilities, uncovering user needs in the development of children’s products becomes even more challenging. User Journey Mapping (UJM) is a visual tool used to analyze the entire process of a user completing a task [11]. In the field of product design, UJM can break down and analyze the user’s product usage process, focusing on their actions, experiences, and pain point issues at each stage. This helps uncover product needs from the user’s perspective [12]. Compared to methods like user interviews or questionnaires, UJM is more suitable for studying the needs of preschool children, as it allows researchers to systematically capture, organize, and grasp children’s needs. User demand analysis and design decision-making are key steps in the product design process. The Analytic Hierarchy Process (AHP) is particularly suitable for handling complex decision-making problems that involve multiple levels and criteria [13]. By constructing a hierarchical structure, AHP breaks down complex needs into several quantifiable indicators and determines the relative weights and priority rankings of these indicators through expert scoring, thus deriving the decision outcome [14]. Compared to other decision-making methods, AHP provides a more precise, scientific, and intuitive analysis process [15], making it easier to understand, grasp, and apply for non-professionals [16]. Due to its clear theoretical logic, ease of understanding and operation, practicality, and systematic nature advantages, AHP has been widely used in the demand analysis and design decisions of various products, such as intelligent wearable masks [17], novel reconfigurable wheelchairs [18], and breastfeeding chairs for maternity rooms [19]. For children’s nebulizer products, since children, parents, and medical staff are all direct or indirect users of the product, the diversity and complexity of user needs make AHP a valuable tool for helping designers analyze user requirements, making the design decision-making process more scientific and well-founded. After guiding design practice based on the results of demands acquisition and analysis, scientific scheme evaluation is also one of the important contents of product design and decision-making research [20]. The fuzzy comprehensive evaluation method (FCE) is an approach that uses fuzzy mathematical principles to conduct overall evaluation analysis of things or evaluation objects [21,22]. It is increasingly applied in design scheme evaluation due to its ability to systematically handle issues that are difficult to quantify or have inherent ambiguity. The FCE method evaluates the factors affecting product quality through fuzzy quantification, comprehensively considers the weights of various factors, and derives a comprehensive evaluation result for the product design scheme [23]. Due to the complexity of pediatric nebulizer design, the FCE method offers a flexible and precise way to assess the feasibility and effectiveness of the proposed solution. Given the complementary strengths of AHP and FCE, their combined application ensures both a rigorous prioritization of design criteria and a robust assessment of user satisfaction. Many scholars have studied the combined application of AHP and FCE in product design. For example, Xie et al. [24] combined AHP and FCE to propose an aesthetic design and evaluation framework for public facilities at train stations, using the inquiry desk at Xiong’an Railway Station as an example for design practice and methodological validation. Zhao et al. [25] explored the design and evaluation of Chinese tea sets using a combination of AHP and FCE, validating the feasibility of the method through the design practice of “The Classic of Mountains and Seas” tea sets. Li et al. [26] established a hierarchical element model for toys designed for children with ADHD based on the AHP-FCE model, calculating the weights of various design indicators to guide the generation of a set of schemes. They then performed a fuzzy comprehensive evaluation on the schemes to select the optimal one. Wang et al. conducted research on the innovative design and scheme evaluation of World Cup cultural and creative lighting using the AHP-FCE model [27]. Sun et al. ranked the design indicators of reusable takeaway containers based on AHP and used FCE to evaluate and optimize the design practice schemes, finally providing design recommendations for reusable takeaway containers [28]. Based on the analysis of the above literature, it can be seen that the AHP-FCE model has been widely applied in product design and evaluation. However, this theoretical method has not yet been applied to the design research of children’s medical nebulizers.

Therefore, this study aims to explore how to systematically design and evaluate children’s medical nebulizer products based on the AHP-FCE method framework, starting from user demands. The goal is to help designers better understand and address the diverse demands of users for children’s medical nebulizers, design products with improved user experience, and promote further development in the field of medical nebulizer design. Additionally, this research seeks to provide scientific theoretical guidance and practical references for the innovative design of children’s medical equipment in the future.

## Materials and methods

### Theoretical overview

#### User journey mapping.

The User Journey Mapping (UJM) is a method commonly used in service design and product user experience design. It visually presents the complete experience of users during their interaction with a product or service [29]. UJM helps researchers and designers break down the stages of user behavior when using a product or service, identifying key interaction points and pain points between the user and the product or service at different stages [30], thus uncovering potential user needs [31]. This method aids in discovering potential improvement opportunities for products [32], optimizing product design and user experience [33].

#### KJ method.

The KJ Method, also known as the Affinity Diagram, is a method for organizing and collating information proposed by a Japanese ethnologist, Jiro Kawakita, in the 1960s [34,35]. This method is centered on collecting relevant facts, opinions, ideas, and other data information about complex problems and using the inherent interrelationships between these data to classify and organize them [36]. It helps teams or researchers to uncover the inherent logical connections and identify the core or potential structure of the issue [37]. The KJ Method is commonly used in areas such as product design, market research, and needs analysis, assisting in the integration of user needs, perspectives, and ideas [38]. It is particularly useful for analyzing and summarizing issues in situations where information is complex and opinions are widely dispersed [39].

#### Analytic hierarchy process.

The Analytic Hierarchy Process (AHP) is a widely used multi-criteria decision-making method developed by American scholar Saaty in the early 1970s [40,41]. AHP breaks down complex decision problems into multiple hierarchical levels [42], where the factors at each level are compared and scored based on their contribution to the decision goal. This allows for the calculation of the weightage values and priority rankings of each evaluation criterion indicator [43]. The strength of AHP lies not only in its ability to handle multi-criteria problems but also in its capacity to integrate both qualitative and quantitative factors, making the decision-making process more scientific, intuitive, and systematic [44]. The AHP typically involves four key steps, as outlined below:

1. Constructing a hierarchical model of demand indicators

Through systematic analysis, complex user demands are organized and summarized into different levels, from the goal level to the criterion level, down to the specific sub-criterion level, forming a structured hierarchical model of demand indicators.

2. Judgment matrix construction

Invite relevant experts to compare the importance of indicators at the same level in the hierarchical model for their upper-level factors, and use the “nine-point scale method” to perform pairwise comparison and scoring to generate judgment matrix A.


$$A={\left(a_{ij}\right)}_{n\times n}$$ (1)

In the formula: i,j=1,2,3,...,n; n represents the order of the matrix; $a_{ij}$ represents the element of the i-th row and j-th column of the matrix;$a_{ij}=\frac{\text{1}}{a_{ij}}$.

3. Calculating the weight vector of each factor using the geometric mean method based on the scoring matrix:

$$\omega_{i}=\frac{{\left[\prod_{j=\text{1}}^{n}a_{ij}\right]}^{\frac{\text{1}}{n}}}{\sum_{i=\text{1}}^{n}{\left[\prod_{j=\text{1}}^{n}a_{ij}\right]}^{\frac{\text{1}}{n}}},i=\text{1},\text{2},\ldots ,n$$ (2)

4. Consistency test. The consistency ratio (CR) of the judgment matrix is calculated to verify the rationality of the scoring. The details are as follows:

Calculate the maximum eigenvalue of the judgment matrix.


$$\lambda_{max}=\frac{\text{1}}{n}\sum_{i=\text{1}}^{n}\frac{(A\omega {)}_{i}}{\omega_{i}}$$ (3)

In the formula, *n* is the number of orders of the judgment matrix; $(A\omega {)}_{\text{i}}$ is the i-th element of the product of matrix A and eigenvector ω.

Calculate the consistency index:


$$CI=\frac{\lambda_{max}-n}{n-\text{1}}$$ (4)

Calculate the consistency ratio:


$$CR=\frac{CI}{RI}$$ (5)

In the formula, RI is the random consistency index, and the RI values of different order matrices are shown in Table 1.

**Table 1:** RI value of matrix order 1-9.

1	2	3	4	5	6	7	8	9
0	0	0.58	0.90	1.12	1.24	1.32	1.41	1.45

If the CR value is less than the permissible threshold of 0.1, the consistency test of the judgment matrix is considered acceptable [45]. If the CR ≥ 0.1, it is necessary to adjust the judgment matrix and re-conduct the consistency test until it is qualified.

#### Fuzzy comprehensive evaluation method.

The Fuzzy Comprehensive Evaluation (FCE) method is an evaluation approach based on fuzzy set theory, proposed by Professor Zadeh in 1965 [46]. FCE quantitatively handles factors that are difficult to quantify or have blurred boundaries using fuzzy mathematics, enabling comprehensive evaluation of complex systems [47]. This method excels in addressing problems characterized by multiple factors, multiple levels, uncertainty, and fuzziness [48]. FCE is commonly used in design and engineering fields to assess the advantages and disadvantages of solutions or to find further optimization directions for a solution, providing scientific evidence for final decision-making [49,50]. The FCE method first establishes the fuzzy set of evaluation factors, then conducts comprehensive calculations by assigning weights and constructing fuzzy evaluation matrices to produce the final evaluation result. The specific steps are as follows:

(1) Establish the comprehensive evaluation indicator factor set V, V={${\text{V}}_{\text{1}},{\text{V}}_{\text{2}},\cdots ,{\text{V}}_{\text{n}}$};
(2) Use Likert’s scale to determine the evaluation levels, create the set of comments X, and assign the corresponding score to each evaluation level.
(3) Establish a fuzzy evaluation matrix R for each factor in the criteria layer. The evaluation result of the i-th sub-criteria layer element included in a certain factor in the criteria layer is represented as ${\text{R}}_{\text{i}}=\{{\text{R}}_{\text{i1}},{\text{R}}_{\text{i2}},\cdots {\text{R}}_{\text{im}}\}$ by a fuzzy set, and the n single-factor evaluation fuzzy sets ${\text{R}}_{\text{1}}$,${\text{R}}_{\text{2}}$,...,${\text{R}}_{\text{n}}$ are used as rows to form a fuzzy comprehensive evaluation matrix R for each factor in the criteria layer, representing the membership degree of the n-th index in the factor set to the m-th level in the evaluation set.

$$R=[\begin{array}{cccc} {\text{R}}_{\text{11}} & {\text{R}}_{\text{12}} & \cdots & {\text{R}}_{\text{1m}}\\ {\text{R}}_{\text{21}} & {\text{R}}_{\text{22}} & \cdots & {\text{R}}_{\text{2m}}\\ \vdots & \vdots & \ddots & \vdots \\ {\text{R}}_{\text{n1}} & {\text{R}}_{\text{n2}} & \cdots & {\text{R}}_{\text{nm}} \end{array}]$$ (6)

(4) After determining the weight vector of each criterion layer indicator, a comprehensive calculation is performed with the corresponding fuzzy evaluation matrix R to obtain the comprehensive evaluation vector T for each criterion layer indicator:

$$\text{T}=\omega \circ \text{R}=(\omega_{\text{1}},\omega_{\text{2}},\cdots ,\omega_{\text{n}})[\begin{array}{cccc} {\text{R}}_{\text{11}} & {\text{R}}_{\text{12}} & \cdots & {\text{R}}_{\text{1m}}\\ {\text{R}}_{\text{21}} & {\text{R}}_{\text{22}} & \cdots & {\text{R}}_{\text{2m}}\\ \vdots & \vdots & \ddots & \vdots \\ {\text{R}}_{\text{n1}} & {\text{R}}_{\text{n2}} & \cdots & {\text{R}}_{\text{nm}} \end{array}]$$ (7)

(5) Calculate the comprehensive evaluation result S of the target layer of the evaluation object, and obtain the final score of the evaluation scheme.

$$S=\omega_{V}\circ T_{V}=\omega_{V}\circ [\begin{array}{c} T_{\text{1}}\\ T_{\text{2}}\\ ...\\ T_{n} \end{array}]$$ (8)

### Design and evaluation process framework for children’s medical nebulizer

This study focuses on the exploration of product design and evaluation methods and does not involve any experimental research related to clinical trials, animals, human tissues, or biological samples. Human participation in this study is limited to field observations and interview surveys. Human participants were recruited based on the principle of voluntary participation in this study. Before the study commenced, the researchers informed all participants about the purpose of the research, the survey process, the use of the collected data, and the participants’ rights, obtaining their consent. All adult participants independently signed the informed consent form. Field observations of children’s nebulization treatment were conducted after obtaining consent from their guardians, who signed an informed consent form. The individual/guardian(s) in this manuscript has given written informed consent (as outlined in PLOS consent form) to publish these case details. The field observation study started on 16, May, 2024, and ended on 24, May, 2024. Interviews with participants in this study were conducted from 16, May, 2024–27, May, 2024. This study does not involve any discussion related to personal religious beliefs, racial identity, political views, sexual orientation, financial information, or any other private matters. All data and information were collected, recorded, and stored anonymously and confidentially, without any actions that would infringe upon the privacy, dignity, health, or human rights of the participants. We confirm that all methods and procedures in this paper were carried out in accordance with relevant guidelines and regulations, adhering to ethical oversight requirements. The results presented in this study are original and have not been published or submitted elsewhere. Thus, this study has been approved by the Anyang Institute of Technology (AYIT) Ethics Review Board.

The design and evaluation process for children’s medical nebulizers proposed in this article can be generally divided into three phases:

#### Phase 1: Collection and Organization of User Demands.

User needs for pediatric nebulizers were gathered through field observations and interviews. Specifically: (i) The User Journey Mapping (UJM) method was used to visually analyze field observation records. Children’s behaviors and emotional responses at interaction touchpoints with the nebulizer were recorded, and pain points were identified through analysis to generate a list of children’s experience demands. (ii) The NVivo 12 software was used to code and analyze interview transcripts, leading to the identification of additional user needs.

#### Phase 2: Construction and Weight Analysis of the Demand Indicator Model.

An expert group categorized demand indicators using the KJ method, and the Analytic Hierarchy Process (AHP) was applied to construct a hierarchical model consisting of goal, criteria, and sub-criteria layers. Experts were then invited to use AHP to determine the weight of each demand indicator.

#### Phase 3: Innovative Design Practice and Solution Comprehensive Evaluation.

The innovative design practice of the preschool children’s medical nebulizer was guided by the weighted results of the demand indicators. The Fuzzy Comprehensive Evaluation (FCE) method was employed to evaluate and compare user satisfaction with the proposed “BreathePlay” nebulizer and an existing widely used nebulizer case to verify the effectiveness of the proposed approach in this research.

**Fig 1:**
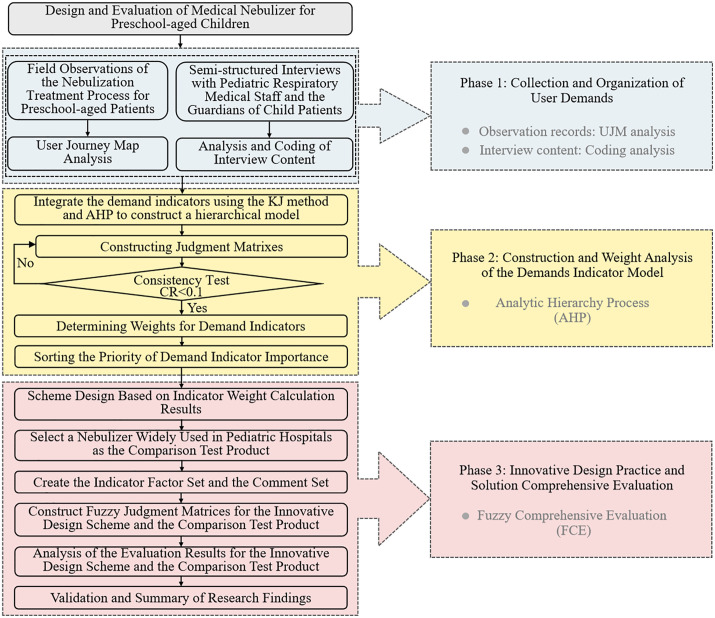
The design and comprehensive evaluation process of preschool-aged children’s medical nebulizer.

The overall design and evaluation process framework is shown in Fig 1.

## Results—Design example of children’s medical nebulizer

### Collection and organization of user demands

#### Selection of the respondent sample.

The stage method of children’s psychological development defines children aged 3–6 as preschool-aged children [51]. Children at this stage mainly rely on the concrete images or representations of things to make judgments, and their cognitive processes are mostly based on their own life experiences or personal emotional judgments, with poor self-control and psychological adjustment abilities [52]. Therefore, preschool-aged children are more prone to developing excessive fear during nebulization therapy due to associations with previous medical experiences, which can trigger strong resistance behaviors. Based on this, the study selected preschool-aged children as the primary target population for research on children’s nebulizers. Children with respiratory diseases are the main service targets of children’s medical atomizers, and they are also the primary group to be considered in the user demand research of this product. Due to children’s physical and psychological immaturity, they cannot seek medical treatment independently and usually need the assistance of their guardians, making guardians also indirect users of children’s medical nebulizers. Additionally, pediatric respiratory medical staff who administer nebulization treatments are the direct operators of the nebulizer, and their operational needs are also critical to the product’s user experience design. Therefore, the samples in this study include preschool-aged children with respiratory diseases, pediatric respiratory medical staff, and children’s guardians, enabling a more comprehensive collection of design demand indicators for preschool-aged children’s medical nebulizers. Considering preschool children’s limited cognitive and verbal expression abilities, their needs were primarily gathered and recorded through field observation, combined with user journey map analysis to identify pain points and demand indicators of children using nebulizers. For pediatric respiratory medical staff and children’s guardians, semi-structured interviews were conducted. Finally, the demand information obtained through different ways was summarized to generate the diverse design demands of the users for children’s medical nebulizers.

Given practical constraints (such as research resources and hospital permissions) and the exploratory nature of the study, a purposive sampling method was used to select participants. The sample size was determined by referencing similar studies and balancing factors such as this study’s objectives, resource limitations, and data saturation [53,54]. This approach ensured a balance between data richness and feasibility [55]. A total of 25 participants were included in the design demand acquisition phase, divided into two groups:

(1) Participant sample for field observation study

10 preschool-aged children receiving nebulization therapy in hospitals (composition of participants: gender: 5 boys, 5 girls; age: 2 children aged 3, 3 children aged 4, 3 children aged 5, and 2 children aged 6; nebulization history: 2 children for the first time, 4 children for 2–3 times, 3 children for 3–5 times, and 1 child for more than 5 times). This group was chosen to identify pain points and needs through direct observation.

(2) Participant sample for interview survey

The interview survey involved 15 participants, including 5 doctors with over 10 years of experience in treating children’s respiratory diseases (2 male, 3 female); 10 guardians who have accompanied children through multiple nebulization treatments (composition of participants: gender: 4 male, 6 female; age range: 1 person aged 20–30, 4 people aged 30–40, 3 people aged 40–50, 2 people over 50; occupation: 2 civil servants, 5 company employees, 1 high school teacher, 1 university lecturer, 1 salesperson; the number of atomization accompaniment experiences: 2 people for less than 3 times, 3 people for 3–5 times, and 5 people for more than 5 times).

The diverse participant composition ensures a comprehensive and multi-perspective understanding of user needs for children’s medical nebulizers, thereby ensuring the validity of the research findings. The sample size and structure were validated through prior literature, ensuring it sufficiently supports the demand analysis.

#### Demand data collection.

Firstly, this study conducted field tracking and observation of 10 selected preschool children (aged 3–6) undergoing treatment with medical nebulizers in a natural state. With prior consent from the children and their guardians, key processes were recorded using photographs and video where appropriate.

Secondly, to ensure that the collected demand information was detailed and comprehensive, one-on-one interviews were conducted with medical staff in pediatric respiratory department and guardians who accompanied their children during nebulization treatment, following pre-prepared interview guidelines (as shown in Tables 2 and 3). During the interviews, researchers encouraged the participants to answer freely without influencing their emotions or leading their responses. Each interview lasted 25–30 minutes. With the participants’ consent, the interview sessions were recorded using audio equipment.

**Table 2:** Interview guideline with pediatric respiratory medical staff.

Serial No.	Open-ended Topic Questions	Probing Questions
1	Do preschool children usually show resistance during nebulization treatment?	If so, what do you think are the reasons for this?
2	Based on your experience with pediatric patients, what do you think are the shortcomings in the appearance design of current medical nebulizers for children?	If the nebulizer’s appearance could be improved, what styling elements or color matching do you think would better meet the needs of preschool children?
3	From your professional perspective, in order to better meet the needs of children’s nebulization treatment, what functional improvements are needed in existing nebulizers?	
4	As medical staff in the pediatric respiratory department, what product issues have affected your experience when operating the nebulizer?	
5	What do you think are the most common difficulties that guardians encounter when using the nebulizer during their child’s nebulization treatment?	
6	What kind of nebulizer design do you think can make children more compliant, comfortable, and enjoyable during the nebulization treatment?	Could you please talk about it with specific experience?

**Table 3:** Interview guideline with guardians of child patients.

Serial No.	Open-ended Topic Questions	Probing Questions
1	Based on your experience, do children often show resistance when using nebulizers for treatment?	(1) If yes, what do you think are the reasons for this? (2) If yes, what methods do you usually use to calm your child’s emotions and make them cooperate with the nebulization treatment?
2	When children use nebulizer products, how do they react to the appearance of the device (such as its color, shape, etc.)?	In your opinion, what kind of appearance design would make children more willing to cooperate with nebulization treatment?
3	When using the nebulizer while accompanying the child during treatment, what difficulties do you face in terms of the nebulizer operation?	
4	During the process of accompanying your child for nebulization treatment, have you noticed any aspects of the nebulizer that are inconvenient or uncomfortable?	Could you share some specific experiences to elaborate?
5	What kind of nebulizer design do you think would make children more cooperative and happier during nebulization treatment?	Could you talk about it in detail?

#### Demand data organization and analysis.

**Fig 2:**
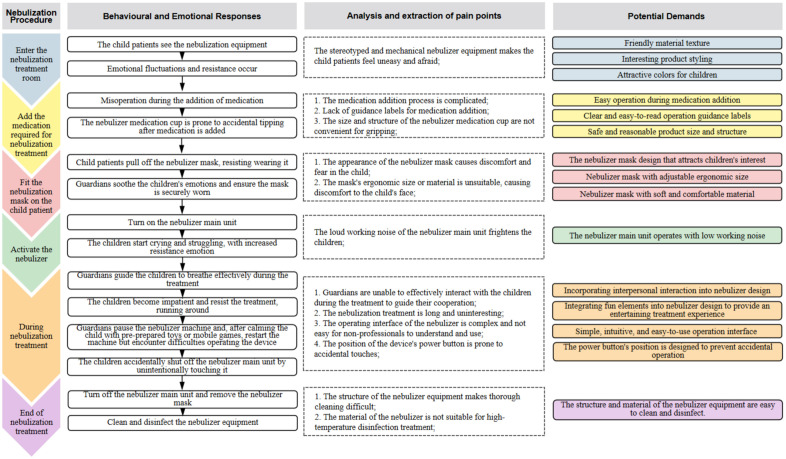
User journey map of the nebulization treatment process in preschool-aged children.

The data analysis in this study includes the following two parts:

Firstly, combine the user journey map with the nebulization treatment flowchart. Based on the field tracking and observation of the entire nebulization treatment process for the child patients, the treatment process is broken down into multiple specific stages. The characteristics of user behaviors in each stage and the factors triggering negative behavioral responses are analyzed. Finally, potential and unknown design needs of users for children’s medical nebulizers are deduced and uncovered based on the above content. The demand extraction and summary based on the user behavior journey map are shown in Fig 2.

Secondly, after the semi-structured interviews with pediatric respiratory doctors and the guardians of child patients, the audio recording materials were transcribed into textual data and analyzed using Nvivo 12 software for coding. To ensure the objectivity and reliability of the interview content coding, and to minimize the impact of subjectivity on the results, this study adopted a cross-coding method involving multiple researchers. Multiple researchers independently coded the same interview data, and then reviewed and compared the processes and results of different coders. Through group discussions and consultations, potential discrepancies in the coding were identified and resolved, ensuring the consistency and accuracy of the coding.

### Construction of a hierarchical model for design indicators of children’s medical nebulizers

**Fig 3:**
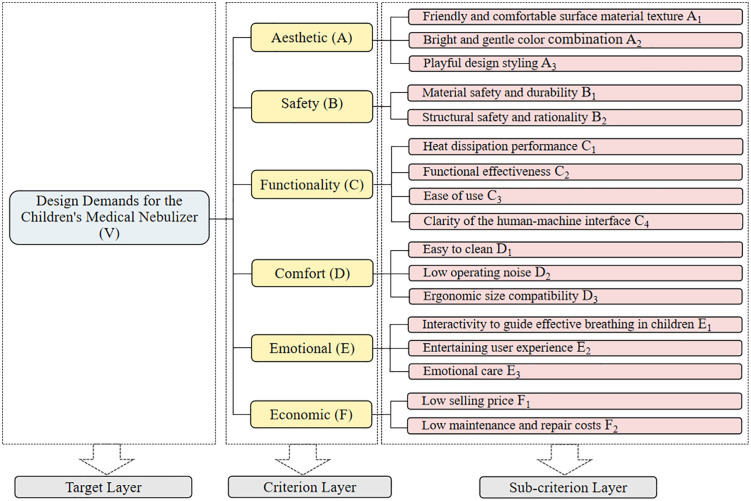
The hierarchical model of design demand indicators for children’s medical nebulizer.

This study invited three designers with more than 10 years of experience in medical product design and two professors in the field of product design to form an expert panel. They used the KJ method to further summarize, organize, and refine the demand indicators derived from the user journey map analysis and the interview content coding. The researchers first recorded each indicator individually on sticky notes. Then, the expert panel clustered and hierarchically divided the indicators based on their differences and correlations, merging or eliminating similar indicators and grouping related items together. Each cluster was assigned a thematic name that encapsulated its primary content. After multiple rounds of screening, splitting, and merging, this study ultimately established a hierarchical structure model of design requirements for children’s medical nebulizers. The model consists of six criteria-level indicators: aesthetics, safety, functionality, comfort, emotional, and economic, along with 17 detailed sub-criteria indicators, as shown in Fig 3.

### Determination of demand indicator weights and priority rankings

#### Constructing a judgment matrix.

Due to the limited cognitive abilities, judgment, and verbal expression skills of preschool children, this study invited experts from relevant fields to provide quantitative ratings for the indicators in the hierarchical model to effectively determine the priority of the importance of demand indicators [56]. sEach expert evaluated and rated the demand indicators in the hierarchical model, drawing on their extensive experience and relevant knowledge in their respective fields. The expert panel used the 1–9 proportional scale method (as shown in Table 4) to pairwise compare and rate the indicators at the same level, ultimately forming judgment matrices of the importance relationships between the indicators [57]. The weight of each indicator was then calculated using Formula (2), with the results shown in Tables 5–12.

**Table 4:** Judgment matrix index importance level numerical scale table.

Scale value	Importance Level	Implication
1	Equally important	Indicator *i* is of equal importance compared to indicator *j*
3	Slightly important	Indicator *i* is slightly important compared to Indicator *j*
5	Obviously important	Indicator *i* is obviously important than Indicator *j*
7	Significantly important	Indicator *i* is significantly important compared to Indicator *j*
9	Absolutely important	Indicator *i* is absolutely important compared to Indicator *j*
2,4,6,8	Intermediate value	The importance level is between two adjacent levels
1/2,1/3...1/9	Reverse comparison	If the importance scale of indicator *i* over indicator *j* is “n”, the reverse comparison is “1/n”.

**Table 5:** Criterion layer judgment matrix and weights.

V	A	B	C	D	E	F	Weights (w)
**A**	1	1/4	1/3	2	1/2	3	0.10347
**B**	4	1	1	5	3	6	0.34770
**C**	3	1	1	3	1	4	0.23689
**D**	1/2	1/5	1/3	1	1/4	3	0.07049
**E**	2	1/3	1	4	1	5	0.20075
**F**	1/3	1/6	1/4	1/3	1/5	1	0.04070

**Table 6:** Judgment matrix and weights for aesthetic criteria.

A	A₁	A₂	A₃	Weights (w)
**A₁**	1	1/2	1/7	0.09774
**A₂**	2	1	1/4	0.18696
**A₃**	7	4	1	0.71530

**Table 7:** Judgment matrix and weights for safety criteria.

B	B₁	B₂	Weights (w)
**B₁**	1	1/2	0.33333
**B₂**	2	1	0.66667

**Table 8:** Judgment matrix and weights for functionality criteria.

C	C₁	C₂	C₃	C4	Weights (w)
**C₁**	1	1/5	1/3	3	0.11907
**C₂**	5	1	2	8	0.53249
**C₃**	3	1/2	1	5	0.29465
**C4**	1/3	1/8	1/5	1	0.05380

**Table 9:** Judgment matrix and weights for comfort criteria.

D	D₁	D₂	D₃	Weights (w)
**D₁**	1	1	1/4	0.16033
**D₂**	1	1	1/5	0.14884
**D₃**	4	5	1	0.69084

**Table 10:** Judgment matrix and weights for emotional criteria.

E	E₁	E₂	E₃	Weights (w)
**E₁**	1	1/3	2	0.23849
**E₂**	3	1	4	0.62501
**E₃**	1/2	1/4	1	0.13650

**Table 11:** Judgment matrix and weights for economic criteria.

F	F₁	F₂	Weights (w)
**F₁**	1	3	0.75000
**F₂**	1/3	1	0.25000

**Table 12:** The comprehensive weights and importance ranking of indicators.

Criterion layer	Weights	Sub-criterion layer	Weights	Comprehensive weights	Ranking
**A**	0.10347	A₁	0.09774	0.01011	17
		A₂	0.18696	0.01934	12
		A₃	0.71530	0.07401	5
**B**	0.34770	B₁	0.33333	0.11590	4
		B₂	0.66667	0.23180	1
**C**	0.23689	C₁	0.11907	0.02821	10
		C₂	0.53249	0.12614	2
		C₃	0.29465	0.06980	6
		C4	0.05380	0.01274	13
**D**	0.07049	D₁	0.16033	0.01130	14
		D₂	0.14884	0.01049	15
		D₃	0.69084	0.04870	7
**E**	0.20075	E₁	0.23849	0.04788	8
		E₂	0.62501	0.12547	3
		E₃	0.13650	0.02740	11
**F**	0.04070	F₁	0.75000	0.03053	9
		F₂	0.25000	0.01018	16

#### Consistency test.

To ensure that the judgments made by the expert panel during the evaluation process are consistent and logical, a consistency test needs to be performed after constructing the judgment matrices [58]. The results calculated according to the steps in formulas 3–5 are shown in Table 13. The CR values of all judgment matrices are less than 0.1, indicating that all the judgment matrices passed the consistency test, and the calculation results are reliable and effective.

**Table 13:** Consistency test results.

Consistency indicators	V	A	B	C	D	E	F
$\lambda_{𝐦𝐚𝐱}$	6.237	3.002	2.000	4.052	3.006	3.018	2.000
**CI**	0.047	0.001	0.000	0.017	0.003	0.009	0.000
**RI**	1.24	0.58	0	0.90	0.58	0.58	0
**CR**	0.038	0.002	0	0.019	0.005	0.016	0

#### Design demands analysis results.

Through the analysis and calculation of demand indicators at various levels for children’s medical nebulizers, the weight values and priority rankings of each indicator were obtained. As shown in the analysis results from Tables 5–12, the highest weighted criterion-layer demand indicator is safety (0.34770), followed by functionality (0.23689), emotional (0.20075), aesthetics (0.10347), comfort (0.07049), and the lowest is economic factors (0.04070). From the overall weight ranking of sub-criterion-layer indicators, the primary demand indicators influencing the design of children’s medical nebulizers are: structural safety and rationality B2 (0.23180)> functional effectiveness C2 (0.12614)> entertaining user experience E2 (0.12547)> material safety and durability B1 (0.11590)> playful design styling A3 (0.07401)> ease of use C3 (0.06980)> ergonomic size compatibility D3 (0.04870)> interactivity to guide effective breathing in children E1 (0.04788).

The results of these demand indicator weights and priority rankings reveal that users’ demands for children’s medical nebulizers have extended beyond just safety and functionality, with increasing emphasis on emotional experience of the product. While safety remains the primary consideration in the design of medical products for children, adding entertainment elements and enhancing interactivity during nebulization to improve the emotional experience is also becoming an important consumer demand. Furthermore, using fun and engaging designs to increase the visual appeal of the nebulizer, thereby alleviating children’s resistance, is another key consideration. Additionally, an easy-to-use operation and ergonomic sizing that caters to children’s physiological needs are also crucial for enhancing the comfort of using the nebulizer. The above demand analysis results can provide valuable references for the subsequent innovative design practice of children’s medical nebulizers.

### Scheme design of children’s medical nebulizer

**Fig 4:**
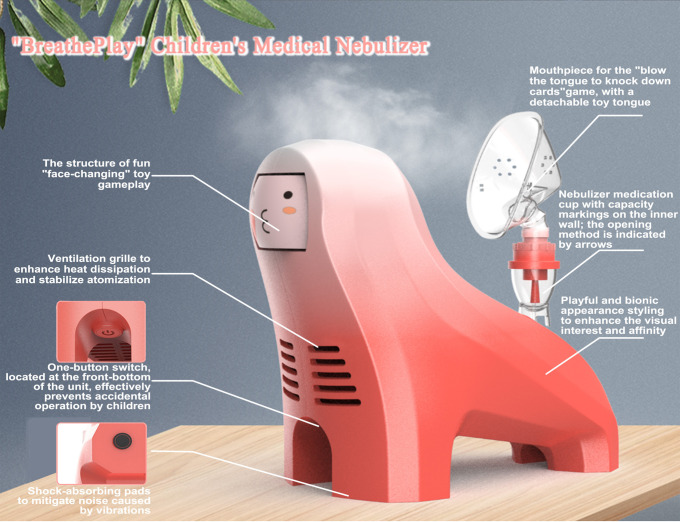
Design rendering 1 of the “BreathePlay” children’s medical nebulizer.

**Fig 5:**
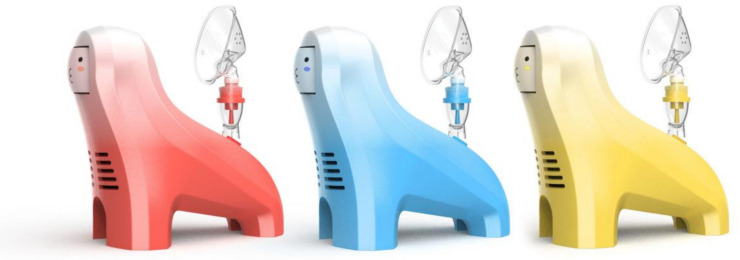
Design rendering 2 of the “BreathePlay” children’s medical nebulizer.

Based on the AHP weight calculations and priority ranking results of the demand indicators, the design concept for the preschool-aged children’s medical nebulizer was conceived. Safety was identified as the highest priority criterion-layer demand indicator, which guides the design considerations in terms of “structural safety and rationality” (weight = 0.23180) and “material safety and durability” (weight = 0.11590). Similarly, the weights of “functional effectiveness (weight =0.12614)” and “entertaining user experience (weight =0.12547)” directed the design of interactive entertainment elements, such as the “blowing tongue” game on the nebulizer mask, which leverages entertaining interaction to enhance deep breathing efficiency and thereby promote the realization of the effectiveness of the nebulization function. Additionally, the demand for “playful design styling” (weight = 0.07401) inspired the anthropomorphic animal-themed appearance design, enhancing visual appeal and reducing children’s anxiety. Finally, the “ease of use” indicator (weight = 0.06980) led to the adoption of a one-button operation and clear medication placement markings to simplify use for caregivers and prevent accidental operation. Table 14 illustrates how the AHP-weighted demand indicators guided the specific design decisions for the “BreathePlay” nebulizer. The innovative design scheme is named “BreathePlay Children’s Medical Nebulizer,” and the final renderings of the solution are shown in Figs 4 and 5.

**Table 14:** Mapping of critical demand indicators and innovative solution design elements.

Critical demand indicators	Design decision considerations	Design implementation
Structural safety and rationality (0.23180); Material safety and durability (0.11590)	Safety Design	Uses safe and comfortable ABS and PP materials; avoids sharp-edged structures.
Functional effectiveness (0.12614); Entertaining user experience E2 (0.12547)	Interactive entertainment design that enhances treatment effectiveness	“Blowing tongue to knock down cards” entertainment game promotes deep breathing for effective treatment; The “face-changing” toy structure is set on the head of the nebulizer host to increase interactive fun.
Playful design styling (0.07401)	Playful design styling to enhance visual appeal	The nebulizer host adopts a fun and bionic appearance to enhance its visual appeal and affinity.
Ease of use (0.06980)	Easy and convenient operation design	one-button operation; adjustable mist output; clear medication placement markers; volume indicators; a child-safe switch placement;

Existing baseline nebulizers primarily focus on functionality, featuring a simple and practical design. However, they fail to meet the psychological and emotional needs of preschool-aged children, which can easily lead to anxiety and resistance toward nebulization therapy. As a result, preschool-aged users may exhibit low therapeutic adherence, making it difficult to ensure effective therapy. Numerous previous studies [59–61] have highlighted the positive impact of incorporating interactive play into medical devices, demonstrating their effectiveness in improving young children’s treatment adherence and reducing anxiety during pediatric treatments. Therefore, the proposed “BreathePlay” children’s medical nebulizer in this study aims to combine visual playful elements with behavioral interactive play methods in accordance with user needs, providing a more comforting emotional experience for children’s nebulization treatment process, thereby improving preschool children’s treatment adherence and concentration. The comparison of usage scenarios between the innovative design of the “BreathePlay” children’s medical nebulizer and the existing nebulizer is shown in Fig 6.

**Fig 6:**
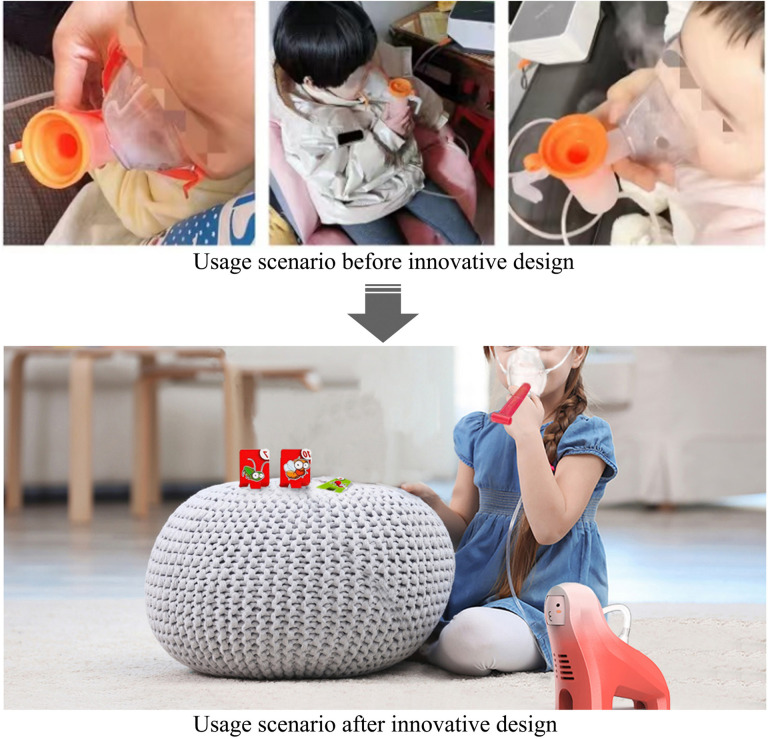
Comparison of usage scenarios before and after the innovative design of the children’s medical nebulizer.

The specific design description for the “BreathePlay Children’s Medical Nebulizer” is as follows:

(1) Safety Design

The safety design in this “BreathePlay” nebulizer scheme addresses both product structure and material safety. First, all parts of the “BreathePlay” nebulizer that are potentially accessible to children are designed to avoid sharp edges to prevent accidental scratches or bumps. Additionally, unlike existing nebulizers, the “BreathePlay” nebulizer features a switch button located at the front-bottom of the unit, effectively preventing accidental operation by children during nebulization therapy. Furthermore, the “BreathePlay” nebulizer adopts ABS for its main body—a material known for its safety, health benefits, and comfortable texture. The medication cup and nebulizer mask are made of PP plastic, meeting the same standards as infant pacifiers, ensuring a comfortable fit for children’s faces, ease of cleaning, and heat sterilization. These improvements help prevent cross-infection of respiratory diseases while enhancing safety and comfort.

(2) Interactive entertainment design that enhances treatment effectiveness

The primary purpose of the children’s medical nebulizer is to provide effective treatment for children with respiratory diseases. Deep breathing helps to deposit nebulized particles in the lungs and lower respiratory tract, allowing the medication to work more effectively. If the child cries during treatment, it will cause their breathing to become shallow and rapid, and the medication is mostly deposited in the mouth rather than reaching the lower respiratory tract, reducing the treatment’s effectiveness. However, it is difficult to get children to follow medical instructions voluntarily. Thus, this design skillfully combines the nebulizer mask with toy entertainment gameplay, promoting children to actively complete effective deep breathing required for treatment during play, alleviating children’s resistance and improving treatment efficacy. The interactive entertainment design of this scheme is implemented in both the nebulizer host and the mask.

**Fig 7:**
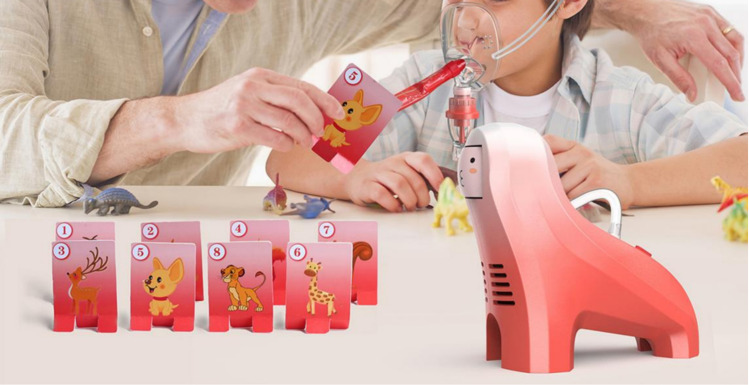
Interactive entertainment way 1 of BreathePlay children’s medical nebulizer.

The “BreathePlay” nebulizer incorporates a detachable “curled toy tongue” in the respiratory mask section (as shown in the “Supporting Information” file S1). The toy tongue is made of a flexible material and has a thin-walled, hollow, tubular shape. When the child exhales, it can be blown out, extended, or straightened, allowing them to play the game of knocking down cards, as shown in Fig 7. Once the exhalation stops, the toy tongue automatically returns to its curled state. During the game of “blowing the tongue”, children need to take deep breaths to store air in order to blow the toy tongue longer and more forcefully to knock down the small cards placed on the table and complete the game. During the process of deep breathing to play the game, effective deep breathing required for children’s nebulization therapy is achieved, promoting the effective realization of nebulization therapy functions. This play method of the child atomizer can also enable children to interact with their parents through games, with parents participating in and guiding the children or collaborating with them to complete the games. Compared to parents directly instructing their children on how to adjust their breathing to complete the treatment, children are more likely to listen to the guidance of parents during interactive games. Parents can more effectively guide their children to adjust their breathing, and parent-child interactive games can also help alleviate children’s anxiety and discomfort during treatment.

**Fig 8:**
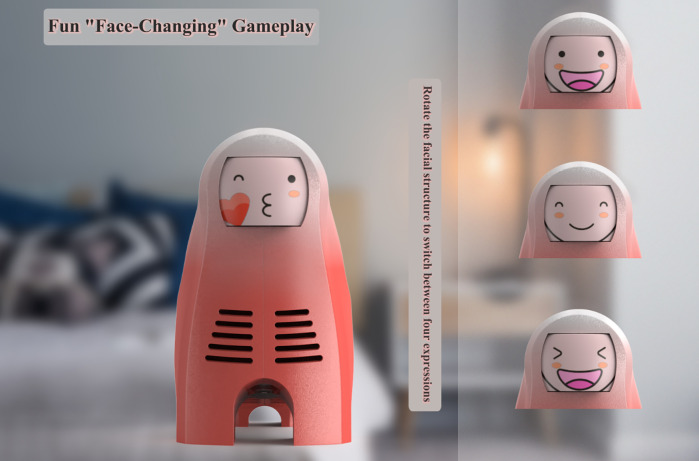
Interactive entertainment way 2 of BreathePlay children’s medical nebulizer.

Additionally, the head of the main body of the “BreathePlay” children’s nebulizer has also been designed with playable structural features, incorporating the fun element of “face changing”. As the design of the children’s atomizer is targeted at preschool children, the entertainment function should not be set too complicated. Therefore, the solution is to set up a “face-changing” small toy on the head of the atomizer host. Children can toggle the facial structure here to change different expressions for the atomizer host, adding novelty and interest, as shown in Fig 8.

(3) Playful design styling to enhance visual appeal

Considering the psychological and cognitive characteristics of preschool children, the design of the children’s nebulizer host in this design scheme adopts a fun and bionic appearance, abstracting the familiar animal images of children. The overall simple, cute, smooth, and vivid shape can enhance the visual interest and affinity of the nebulizer product. This reduces the emotional distance between children and the medical device, helping to alleviate resistance and anxiety.

(4) Easy and convenient operation design

The “BreathePlay” children’s nebulizer is designed for ease of use with a one-button switch, making it simple and user-friendly. The mist output can be freely adjusted according to need. The medication placement area is marked with an arrow to indicate how to open it, and the medication cup includes volume markers to make it easy for medical staff or parents to understand and use.

### Scheme verification

**Fig 9:**
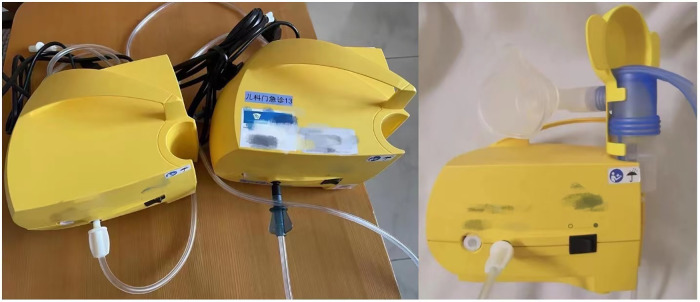
A nebulizer widely used in pediatric hospitals as a comparison product.

To validate the feasibility and user satisfaction of the “BreathePlay” children’s medical nebulizer innovative design scheme, this study produced a prototype of the design and applied the fuzzy comprehensive evaluation method to assess the design scheme. Additionally, this study incorporated the opinions of several experienced pediatric respiratory doctors and selected a nebulizer widely used in pediatric hospitals as a comparison product (see Fig 9), to verify whether the comprehensive performance of the “BreathePlay” nebulizer design scheme has effectively improved compared to the existing product.

A Fuzzy Comprehensive Evaluation questionnaire was developed based on the demand indicator hierarchy model for children’s medical nebulizer design (for details, see “Supporting Information” S2 File). Each questionnaire consisted of two pages: Page A focused on the evaluation of the “BreathePlay” innovative design product, and Page B focused on the evaluation of the selected comparison nebulizer product. The evaluation indicators from the hierarchy model were used as the factor set V, $\text{V}=\left\{{\text{V}}_{\text{A}},{\text{V}}_{\text{B}},{\text{V}}_{\text{C}},{\text{V}}_{\text{D}},{\text{V}}_{\text{E}},{\text{V}}_{\text{F}}\right\}$, and a five-point Likert scale was employed to establish the comment set X, X ={Very Satisfied, Satisfied, Average, Dissatisfied, Very Dissatisfied}. Different comment levels were assigned corresponding scores, and the evaluation vector after assignment is $\beta ={\left\{\text{90},\text{80},\text{70},\text{60},\text{50}\right\}}^{\text{T}}$[62].

The user questionnaire survey was conducted in-person, and the respondents mainly included guardians with experience in supervising children’s nebulization treatments, as well as medical staff in the pediatric respiratory department. The researchers first invited the respondents who were about to receive this survey to use both the prototype of the “BreathePlay” innovative design and the selected existing nebulizer product. Then, they explained the meaning of the demand indicators and evaluation levels in detail to the respondents. Finally, the respondents officially filled out the evaluation questionnaire. A total of 97 valid questionnaires were collected in this survey, and the survey results are detailed in the “Supporting Information” S3 File.

The number of times respondents rated each demand indicator in each evaluation level was tallied, leading to the derivation of the membership degree of each evaluation indicator relative to each evaluation level. Based on this, the fuzzy comprehensive evaluation matrices R were constructed for the aesthetic (A), safety (B), functionality (C), comfort (D), emotional (E), and economic (F) aspects of the children’s medical nebulizer innovative design:


$$R_{A}=[\begin{array}{ccccc} \text{0}.\text{2}\text{8}\text{9} & \text{0}.\text{6}\text{2}\text{9} & \text{0}.\text{0}\text{8}\text{2} & \text{0}.\text{0}\text{0}\text{0} & \text{0}.\text{0}\text{0}\text{0}\\ \text{0}.\text{2}\text{1}\text{6} & \text{0}.\text{5}\text{2}\text{6} & \text{0}.\text{2}\text{4}\text{7} & \text{0}.\text{0}\text{1}\text{0} & \text{0}.\text{0}\text{0}\text{0}\\ \text{0}.\text{5}\text{9}\text{8} & \text{0}.\text{2}\text{7}\text{8} & \text{0}.\text{1}\text{2}\text{4} & \text{0}.\text{0}\text{0}\text{0} & \text{0}.\text{0}\text{0}\text{0} \end{array}]$$

$$R_{B}=[\begin{array}{ccccc} \text{0}.\text{7}\text{6}\text{3} & \text{0}.\text{2}\text{1}\text{6} & \text{0}.\text{0}\text{2}\text{1} & \text{0}.\text{0}\text{0}\text{0} & \text{0}.\text{0}\text{0}\text{0}\\ \text{0}.\text{7}\text{1}\text{1} & \text{0}.\text{1}\text{9}\text{6} & \text{0}.\text{0}\text{9}\text{3} & \text{0}.\text{0}\text{0}\text{0} & \text{0}.\text{0}\text{0}\text{0} \end{array}]$$

$$R_{C}=[\begin{array}{ccccc} \text{0}.\text{2}\text{6}\text{8} & \text{0}.\text{7}\text{0}\text{1} & \text{0}.\text{0}\text{3}\text{1} & \text{0}.\text{0}\text{0}\text{0} & \text{0}.\text{0}\text{0}\text{0}\\ \text{0}.\text{7}\text{9}\text{4} & \text{0}.\text{1}\text{9}\text{6} & \text{0}.\text{0}\text{1}\text{0} & \text{0}.\text{0}\text{0}\text{0} & \text{0}.\text{0}\text{0}\text{0}\\ \text{0}.\text{3}\text{9}\text{2} & \text{0}.\text{5}\text{8}\text{8} & \text{0}.\text{0}\text{2}\text{1} & \text{0}.\text{0}\text{0}\text{0} & \text{0}.\text{0}\text{0}\text{0}\\ \text{0}.\text{2}\text{3}\text{7} & \text{0}.\text{5}\text{9}\text{8} & \text{0}.\text{1}\text{6}\text{5} & \text{0}.\text{0}\text{0}\text{0} & \text{0}.\text{0}\text{0}\text{0} \end{array}]$$

$$R_{D}=[\begin{array}{ccccc} \text{0}.\text{1}\text{7}\text{5} & \text{0}.\text{5}\text{4}\text{6} & \text{0}.\text{2}\text{5}\text{8} & \text{0}.\text{0}\text{2}\text{1} & \text{0}.\text{0}\text{0}\text{0}\\ \text{0}.\text{1}\text{4}\text{4} & \text{0}.\text{5}\text{2}\text{6} & \text{0}.\text{2}\text{9}\text{9} & \text{0}.\text{0}\text{3}\text{1} & \text{0}.\text{0}\text{0}\text{0}\\ \text{0}.\text{4}\text{2}\text{3} & \text{0}.\text{4}\text{6}\text{4} & \text{0}.\text{1}\text{1}\text{3} & \text{0}.\text{0}\text{0}\text{0} & \text{0}.\text{0}\text{0}\text{0} \end{array}]$$

$$R_{E}=[\begin{array}{ccccc} \text{0}.\text{2}\text{7}\text{8} & \text{0}.\text{5}\text{7}\text{7} & \text{0}.\text{1}\text{4}\text{4} & \text{0}.\text{0}\text{0}\text{0} & \text{0}.\text{0}\text{0}\text{0}\\ \text{0}.\text{8}\text{1}\text{4} & \text{0}.\text{1}\text{8}\text{6} & \text{0}.\text{0}\text{0}\text{0} & \text{0}.\text{0}\text{0}\text{0} & \text{0}.\text{0}\text{0}\text{0}\\ \text{0}.\text{3}\text{3}\text{0} & \text{0}.\text{3}\text{8}\text{1} & \text{0}.\text{2}\text{8}\text{9} & \text{0}.\text{0}\text{0}\text{0} & \text{0}.\text{0}\text{0}\text{0} \end{array}]$$

$$R_{F}=[\begin{array}{ccccc} \text{0}.\text{0}\text{9}\text{3} & \text{0}.\text{3}\text{2}\text{0} & \text{0}.\text{5}\text{5}\text{7} & \text{0}.\text{0}\text{3}\text{1} & \text{0}.\text{0}\text{0}\text{0}\\ \text{0}.\text{2}\text{6}\text{8} & \text{0}.\text{5}\text{0}\text{5} & \text{0}.\text{2}\text{2}\text{7} & \text{0}.\text{0}\text{0}\text{0} & \text{0}.\text{0}\text{0}\text{0} \end{array}]$$

Using the weighted average fuzzy operator, the fuzzy evaluation matrix was combined with the weights obtained from the analytic hierarchy process to calculate the evaluation weight vector for each criterion-level indicator as follows:


$$T_{A}=\omega_{A}\circ R_{A}=(\text{0}.\text{4}\text{9}\text{6}\text{0}.\text{3}\text{5}\text{9}\text{0}.\text{1}\text{4}\text{3}\text{0}.\text{0}\text{0}\text{2}\text{0}.\text{0}\text{0}\text{0})$$

$$T_{B}=\omega_{B}\circ R_{B}=(\text{0}.\text{7}\text{2}\text{8}\text{0}.\text{2}\text{0}\text{3}\text{0}.\text{0}\text{6}\text{9}\text{0}.\text{0}\text{0}\text{0}\text{0}.\text{0}\text{0}\text{0})$$

$$T_{C}=\omega_{C}\circ R_{C}=(\text{0}.\text{5}\text{8}\text{3}\text{0}.\text{3}\text{9}\text{3}\text{0}.\text{0}\text{2}\text{4}\text{0}.\text{0}\text{0}\text{0}\text{0}.\text{0}\text{0}\text{0})$$

$$T_{D}=\omega_{D}\circ R_{D}=(\text{0}.\text{3}\text{4}\text{2}\text{0}.\text{4}\text{8}\text{6}\text{0}.\text{1}\text{6}\text{4}\text{0}.\text{0}\text{0}\text{8}\text{0}.\text{0}\text{0}\text{0})$$

$$T_{E}=\omega_{E}\circ R_{E}=(\text{0}.\text{6}\text{2}\text{0}\text{0}.\text{3}\text{0}\text{6}\text{0}.\text{0}\text{7}\text{4}\text{0}.\text{0}\text{0}\text{0}\text{0}.\text{0}\text{0}\text{0})$$

$$T_{F}=\omega_{F}\circ R_{F}=(\text{0}.\text{1}\text{3}\text{7}\text{0}.\text{3}\text{6}\text{6}\text{0}.\text{4}\text{7}\text{4}\text{0}.\text{0}\text{2}\text{3}\text{0}.\text{0}\text{0}\text{0})$$

On this basis, the comprehensive evaluation vector S for the target layer of the children’s medical nebulizer design scheme was calculated as follows:


$$S=\omega_{V}\circ T_{V}=\omega_{V}\circ [\begin{array}{c} T_{A}\\ T_{B}\\ T_{C}\\ T_{D}\\ T_{E}\\ T_{F} \end{array}]=(\text{0}.\text{5}\text{9}\text{7}\text{0}.\text{3}\text{1}\text{1}\text{0}.\text{0}\text{9}\text{0}\text{0}.\text{0}\text{0}\text{2}\text{0})$$

Finally, through weighted calculation between the comprehensive evaluation vector S and the corresponding scores of the comment set levels, the score for the “BreathePlay” children’s medical nebulizer design scheme on a 100-point scale is obtained as N = 85.03.

**Fig 10:**
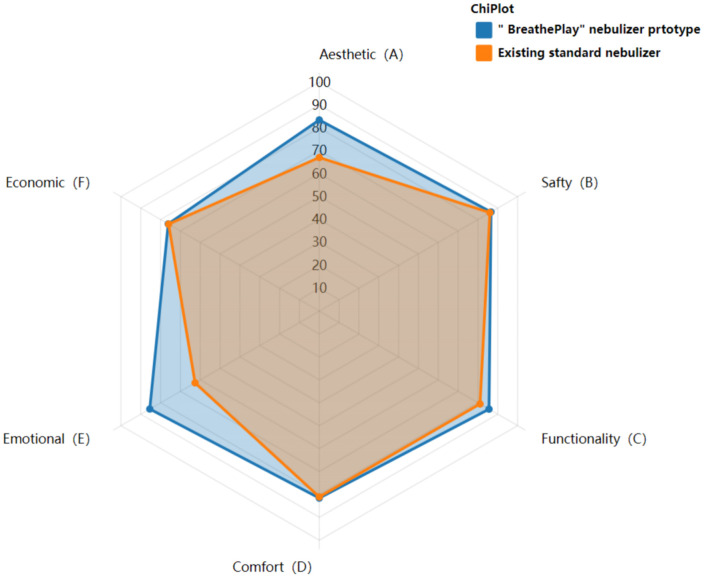
Radar chart of fuzzy comprehensive evaluation results.

Based on the correspondence between the fuzzy evaluation grades and scores, the user satisfaction level for the “BreathePlay” children’s medical nebulizer innovative design is rated as “satisfied,” indicating that the design overall meets users’ diverse needs well. Similarly, following the same steps, the evaluation score for the selected existing nebulizer product case was calculated to be 77.48 points (detailed calculation data can be found in the “Supporting Information” S4 File), with a satisfaction level of “average.” Combining these evaluation results, it can be concluded that the overall satisfaction of the “BreathePlay” innovative design proposed in this study is higher than that of the currently widely used children’s nebulizer products, as shown in Fig 10. Specifically, in terms of emotional criteria (entertaining user experience, interactivity to guide effective breathing in children, and emotional care) and aesthetic criteria (playful design styling), the user satisfaction with the innovative design proposed in this study has significantly improved compared to the existing nebulizer product case. Thus, these results strongly validate the feasibility and effectiveness of this study.

## Conclusion and discussion

Existing research on medical nebulizers primarily focuses on improving product performance and enhancing technical specialization. However, there is still a gap in the study of specialized nebulizer products and design evaluation methods that address the diverse physiological and psychological needs of preschool children. Thus, this study explores an innovative design and evaluation framework for children’s medical nebulizers. By integrating various research methods such as field observations, user journey mapping, and semi-structured interviews, this study systematically and comprehensively analyzes the diverse and potential needs of users regarding children’s medical nebulizers. Using the KJ method, the study screened, supplemented, clustered, and categorized demand indicators, eventually constructing a hierarchical model of design demand indicators for children’s medical nebulizers. This model provides a reference for future design and evaluation of children’s medical nebulizers. On the basis of this demand indicator hierarchy model, the study applied the Analytic Hierarchy Process (AHP) to calculate the weights and prioritize each demand indicator. The results reveal that in addition to safety and functionality, factors such as entertaining user experience, interactivity to guide effective breathing in children, ease of use, and playful design styling carry significant weight in the design of children’s nebulizers. The determination of these key demand indicators guides the formulation of the design scheme in this study and offers a clear direction for future designers to develop innovative children’s nebulizers that better meet user needs. Building on the AHP analysis results, the study proceeded to conduct an innovative design practice for children’s medical nebulizers. Finally, the Fuzzy Comprehensive Evaluation (FCE) method was employed to assess the satisfaction level of the comprehensive performance of the “BreathePlay” innovative design scheme. The evaluation results show that the design of the “BreathePlay” nebulizer can better meet user needs across various dimensions, and its overall satisfaction level significantly surpasses that of the currently widely used children’s nebulizer product. This fully verifies the applicability and effectiveness of the AHP-FCE model in the design of children’s medical nebulizers. Beyond nebulizers, this methodological framework could be adapted to the design of other pediatric devices, such as inhalers or oxygen therapy devices. For instance, incorporating interactive play elements in inhalers might encourage proper inhalation techniques among young children, while a playful oxygen therapy interface could reduce anxiety and improve treatment adherence. Future research could explore how these methods can be effectively tailored to different pediatric medical products. In summary, this study provides a systematic and scientific framework for the design and evaluation of children’s medical nebulizers by combining UJM, KJ, AHP, and FCE methods. This framework ensures comprehensive capture of design needs, precise alignment of design directions with user demands, and thorough evaluation of design solutions. The results contribute to the development of children’s nebulizer products that better meet user expectations. This research not only enriches the theoretical foundations in the field of children’s medical device design but also offers valuable references for the design and development of similar products in the future. The promotion and application of the study’s findings will contribute to innovative development in the children’s medical nebulizer industry, supporting efforts to address pediatric respiratory health issues. Hence, this study holds significant theoretical, practical, and social value.

Despite these contributions, this study has certain limitations. Firstly, while the playful design significantly enhances user compliance, further research could explore additional interaction methods to maintain engagement over repeated uses. For example, incorporating a dynamic reward system or personalized game mechanics may help sustain children’s interest over prolonged treatment periods. Additionally, while this study evaluates user experience and preliminary effectiveness, further clinical validation is needed to assess therapeutic outcomes of the design. The “crimped toy tongue” on the breathing mask in the design practice solution, while designed to engage children during nebulization therapy, may have potential limitations. It might lead to forceful exhalation, potentially reducing drug deposition in the airways. This possible limitation highlights the need for further investigation. Future studies could conduct more rigorous experimental tests based on radiolabelled drug analysis to assess the drug delivery effectiveness of this newly designed nebulizer compared to standard nebulizers, thereby further improving the rigor of the design. Secondly, during the demand research phase, due to the special nature of the hospital environment and the inconvenience for medical personnel and patients to participate in interviews or follow-up observations, the number of participants in this study’s field observations and interviews is limited, and the results obtained may have certain constraints. Furthermore, the research sample of this study was primarily concentrated in Henan Province, China, and may not fully represent the needs of users in all regions or sociocultural contexts. Different cultural backgrounds, healthcare policies, and medical infrastructures may influence both the design expectations of children’s medical devices and the user experience. Sociocultural attitudes toward pediatric care, levels of health literacy, and economic factors could shape user preferences differently. To enhance the generalizability of the findings, future research could further expand the sample size and include participants from diverse geographic and cultural backgrounds. A comparative study across different regions or healthcare systems (e.g., developed vs. developing countries, public vs. private hospitals) would provide deeper insights into how varying contexts influence design needs and validate the framework’s broader applicability.

## Supporting information

S1 File The normal curled state VS the blown and extended state of the toy tongue. (PDF)

S2 File Fuzzy comprehensive evaluation questionnaire. (PDF)

S3 File Statistical results of questionnaire survey data. (PDF)

S4 File Calculate data supplements. (PDF)

S5 File Inclusivity-in-global-research-questionnaire. (PDF)
