# Supplementary material for: Innovative design and evaluation of medical nebulizer for preschool children: A user demand-driven approach
Source: PLoS One. 2025 Dec 1;20(12):e0325199. doi: 10.1371/journal.pone.0325199 (PMC12668560; doi:10.1371/journal.pone.0325199)
Supplement: S1 File — (PDF) [file pone.0325199.s001.pdf]

The normal curled state VS the blown and extended state of the "toy tongue"

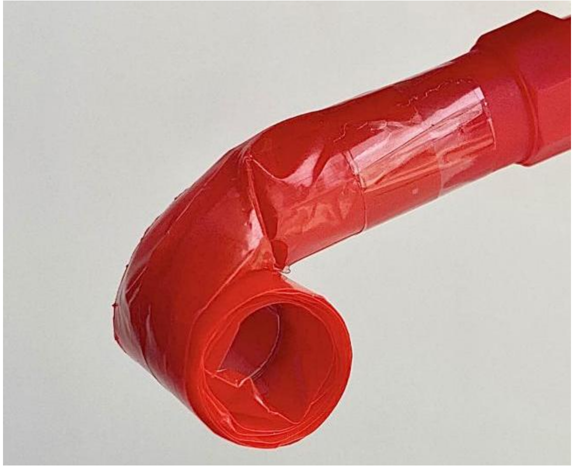

The normal curled state of a detachable "curled toy tongue" designed in the respiratory mask part of the "BreathePlay" nebulizer.

(The toy tongue features a thin-walled hollow tubular shape and is made of flexible material. When the child exhales, the "curled toy tongue" can be blown out, extended, or straightened, and it automatically returns to its curled state when exhaling stops.)

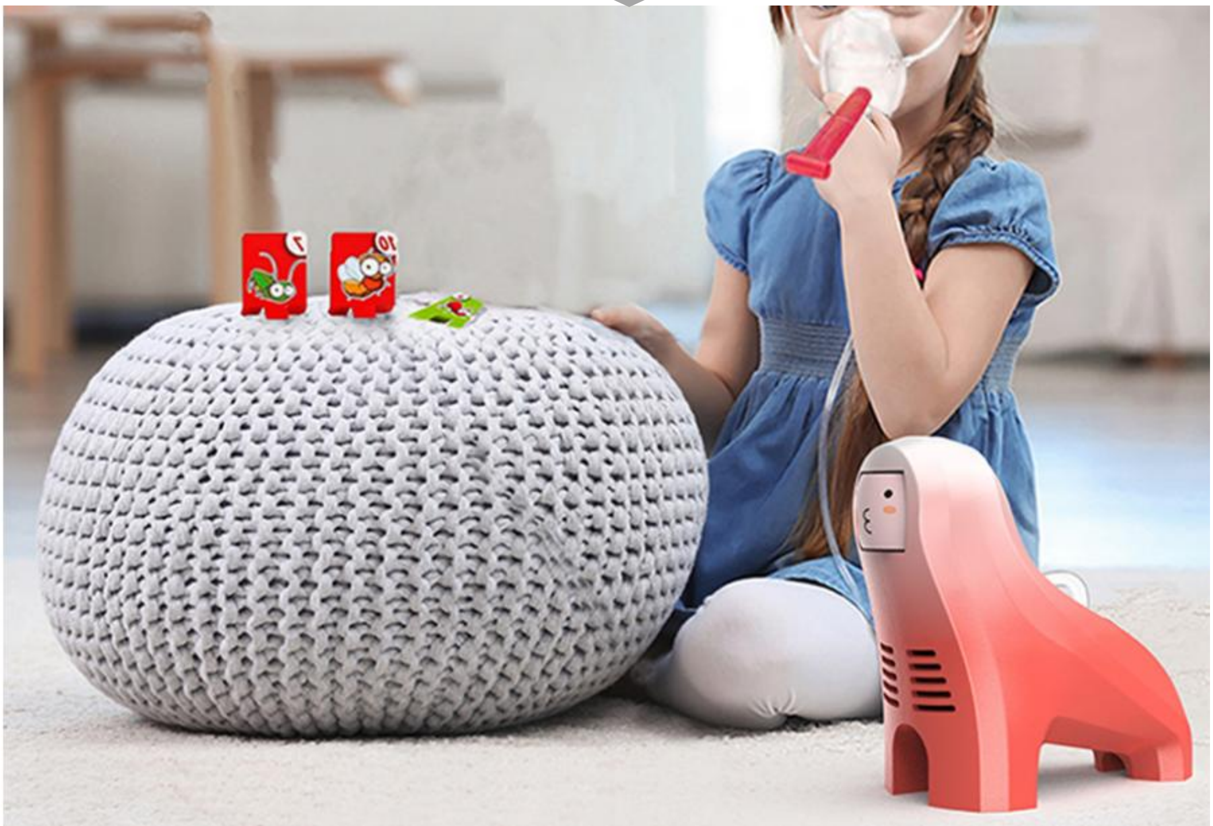

When the child exhales, the "curled toy tongue" can be blown out, straightened, and elongated, allowing them to play the knockdown card game.

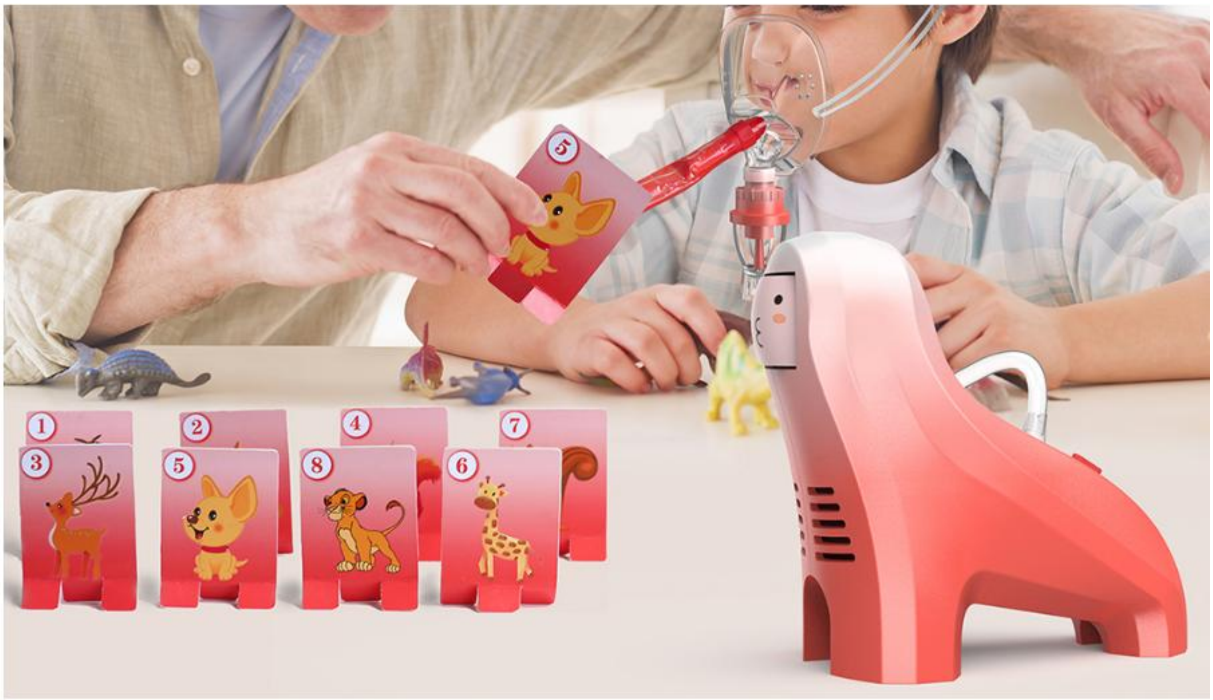

When the child exhales, the "curled toy tongue" can be blown out, straightened, and elongated, allowing them to play the knockdown card game.
