# Supplementary material for: Innovative design and evaluation of medical nebulizer for preschool children: A user demand-driven approach
Source: PLoS One. 2025 Dec 1;20(12):e0325199. doi: 10.1371/journal.pone.0325199 (PMC12668560; doi:10.1371/journal.pone.0325199)
Supplement: S2 File — (PDF) [file pone.0325199.s002.pdf]

Questionnaire on user satisfaction with the 'BreathePlay' children's medical nebulizer

Evaluation indicators	Comment levels
Friendly and comfortable surface material texture (A_1)	□Very Satisfied □Satisfied □Average □Dissatisfied □Very Dissatisfied
Bright and gentle color combination (A_2)	□Very Satisfied □Satisfied □Average □Dissatisfied □Very Dissatisfied
Playful design styling (A_3)	□Very Satisfied □Satisfied □Average □Dissatisfied □Very Dissatisfied
Material safety and durability (B_1)	□Very Satisfied □Satisfied □Average □Dissatisfied □Very Dissatisfied
Structural safety and rationality (B_2)	□Very Satisfied □Satisfied □Average □Dissatisfied □Very Dissatisfied
Heat dissipation performance (C_1)	□Very Satisfied □Satisfied □Average □Dissatisfied □Very Dissatisfied
Functional effectiveness (C_2)	□Very Satisfied □Satisfied □Average □Dissatisfied □Very Dissatisfied
Ease of use (C_3)	□Very Satisfied □Satisfied □Average □Dissatisfied □Very Dissatisfied
Clarity of the human-machine interface (C_4)	□Very Satisfied □Satisfied □Average □Dissatisfied □Very Dissatisfied
Easy to clean (D_1)	□Very Satisfied □Satisfied □Average □Dissatisfied □Very Dissatisfied
Low operating noise (D_2)	□Very Satisfied □Satisfied □Average □Dissatisfied □Very Dissatisfied
Ergonomic size compatibility (D_3)	□Very Satisfied □Satisfied □Average □Dissatisfied □Very Dissatisfied
Interactivity to guide effective breathing in children (E_1)	□Very Satisfied □Satisfied □Average □Dissatisfied □Very Dissatisfied
Entertaining user experience (E_2)	□Very Satisfied □Satisfied □Average □Dissatisfied □Very Dissatisfied
Emotional care (E_3)	□Very Satisfied □Satisfied □Average □Dissatisfied □Very Dissatisfied
Low selling price (F_1)	□Very Satisfied □Satisfied □Average □Dissatisfied □Very Dissatisfied
Low maintenance and repair costs (F_2)	□Very Satisfied □Satisfied □Average □Dissatisfied □Very Dissatisfied

Questionnaire on user satisfaction with the selected existing nebulizer product

Evaluation indicators	Comment levels
Friendly and comfortable surface material texture (A_1)	□Very Satisfied □Satisfied □Average □Dissatisfied □Very Dissatisfied
Bright and gentle color combination (A_2)	□Very Satisfied □Satisfied □Average □Dissatisfied □Very Dissatisfied
Playful design styling (A_3)	□Very Satisfied □Satisfied □Average □Dissatisfied □Very Dissatisfied
Material safety and durability (B_1)	□Very Satisfied □Satisfied □Average □Dissatisfied □Very Dissatisfied
Structural safety and rationality (B_2)	□Very Satisfied □Satisfied □Average □Dissatisfied □Very Dissatisfied
Heat dissipation performance (C_1)	□Very Satisfied □Satisfied □Average □Dissatisfied □Very Dissatisfied
Functional effectiveness (C_2)	□Very Satisfied □Satisfied □Average □Dissatisfied □Very Dissatisfied
Ease of use (C_3)	□Very Satisfied □Satisfied □Average □Dissatisfied □Very Dissatisfied
Clarity of the human-machine interface (C_4)	□Very Satisfied □Satisfied □Average □Dissatisfied □Very Dissatisfied
Easy to clean (D_1)	□Very Satisfied □Satisfied □Average □Dissatisfied □Very Dissatisfied
Low operating noise (D_2)	□Very Satisfied □Satisfied □Average □Dissatisfied □Very Dissatisfied
Ergonomic size compatibility (D_3)	□Very Satisfied □Satisfied □Average □Dissatisfied □Very Dissatisfied
Interactivity to guide effective breathing in children (E_1)	□Very Satisfied □Satisfied □Average □Dissatisfied □Very Dissatisfied
Entertaining user experience (E_2)	□Very Satisfied □Satisfied □Average □Dissatisfied □Very Dissatisfied
Emotional care (E_3)	□Very Satisfied □Satisfied □Average □Dissatisfied □Very Dissatisfied
Low selling price (F_1)	□Very Satisfied □Satisfied □Average □Dissatisfied □Very Dissatisfied
Low maintenance and repair costs (F_2)	□Very Satisfied □Satisfied □Average □Dissatisfied □Very Dissatisfied
