# Supplementary material for: Innovative design and evaluation of medical nebulizer for preschool children: A user demand-driven approach
Source: PLoS One. 2025 Dec 1;20(12):e0325199. doi: 10.1371/journal.pone.0325199 (PMC12668560; doi:10.1371/journal.pone.0325199)
Supplement: S3 File — (PDF) [file pone.0325199.s003.pdf]

S2. Statistical Results of Questionnaire Survey Data

Satisfaction survey data statistics for the 'BreathePlay' Children's Medical Nebulizer

Criterion layer	Sub-criterion layer	Very Satisfied	Satisfied	Average	Dissatisfied	Very Dissatisfied
Aesthetic (A)	Friendly and comfortable surface material texture (A_1)	28	61	8	0	0
	Bright and gentle color combination (A_2)	21	51	24	1	0
	Playful design styling (A_3)	58	27	12	0	0
Safety (B)	Material safety and durability (B_1)	74	21	2	0	0
	Structural safety and rationality (B_2)	69	19	9	0	0
Functionality (C)	Heat dissipation performance (C_1)	26	68	3	0	0
	Functional effectiveness (C_2)	77	19	1	0	0
	Ease of use (C_3)	38	57	2	0	0
	Clarity of the human-machine interface (C_4)	23	58	16	0	0
Comfort (D)	Easy to clean (D_1)	17	53	25	2	0
	Low operating noise (D_2)	14	51	29	3	0
	Ergonomic size compatibility (D_3)	41	45	11	0	0
Emotional (E)	Interactivity to guide effective breathing in children (E_1)	27	56	14	0	0
	Entertaining user experience (E_2)	79	18	0	0	0
	Emotional care (E_3)	32	37	28	0	0
Economic (F)	Low selling price (F_1)	9	31	54	3	0
	Low maintenance and repair costs (F_2)	26	49	22	0	0

Satisfaction survey data statistics for the selected existing nebulizer product

Criterion layer	Sub-criterion layer	Very Satisfied	Satisfied	Average	Dissatisfied	Very Dissatisfied
Aesthetic (A)	Friendly and comfortable surface material texture (A_1)	23	63	11	0	0
	Bright and gentle color combination (A_2)	19	49	26	3	0
	Playful design styling (A_3)	0	6	21	67	3
Safety (B)	Material safety and durability (B_1)	76	16	5	0	0
	Structural safety and rationality (B_2)	66	17	14	0	0
Functionality (C)	Heat dissipation performance (C_1)	11	33	48	4	0
	Functional effectiveness (C_2)	31	49	17	0	0
	Ease of use (C_3)	32	61	4	0	0
	Clarity of the human-machine interface (C_4)	14	53	30	0	0
Comfort (D)	Easy to clean (D_1)	21	48	26	2	0
	Low operating noise (D_2)	11	44	26	16	0
	Ergonomic size compatibility (D_3)	40	45	12	0	0

Emotional (E)	Interactivity to guide effective breathing in children (E_1)	0	2	19	74	2
	Entertaining user experience (E_2)	0	3	23	71	0
	Emotional care (E_3)	1	12	26	55	3
Economic (F)	Low selling price (F_1)	8	33	49	7	0
	Low maintenance and repair costs (F_2)	27	52	18	0	0
