# Supplementary material for: Innovative design and evaluation of medical nebulizer for preschool children: A user demand-driven approach
Source: PLoS One. 2025 Dec 1;20(12):e0325199. doi: 10.1371/journal.pone.0325199 (PMC12668560; doi:10.1371/journal.pone.0325199)
Supplement: S4 File — (PDF) [file pone.0325199.s004.pdf]

The following is the detailed process for calculating the final evaluation score of the selected existing nebulizer product (Figure 9), which is widely used in children's hospitals, through the fuzzy comprehensive evaluation method:

The number of times respondents rated each demand indicator in each evaluation level was tallied, leading to the derivation of the membership degree of each evaluation indicator relative to each evaluation level. Based on this, the fuzzy comprehensive evaluation matrices R were constructed for the aesthetic (A), safety (B), functionality (C), comfort (D), emotional (E), and economic (F) aspects of the selected existing nebulizer product:

$$R_A=\begin{bmatrix}0.237 & 0.649 & 0.113 & 0.000 & 0.000\\ 0.196 & 0.505 & 0.268 & 0.031 & 0.000\\ 0.000 & 0.062 & 0.216 & 0.691 & 0.031\end{bmatrix}$$

$$R_B=\begin{bmatrix}0.784 & 0.165 & 0.052 & 0.000 & 0.000\\ 0.680 & 0.175 & 0.144 & 0.000 & 0.000\end{bmatrix}$$

$$R_C=\begin{bmatrix}0.113 & 0.340 & 0.495 & 0.041 & 0.000\\ 0.320 & 0.505 & 0.175 & 0.000 & 0.000\\ 0.330 & 0.629 & 0.041 & 0.000 & 0.000\\ 0.144 & 0.546 & 0.309 & 0.000 & 0.000\end{bmatrix}$$

$$R_D=\begin{bmatrix}0.216 & 0.495 & 0.268 & 0.021 & 0.000\\ 0.113 & 0.454 & 0.268 & 0.165 & 0.000\\ 0.412 & 0.464 & 0.124 & 0.000 & 0.000\end{bmatrix}$$

$$R_E=\begin{bmatrix}0.000 & 0.021 & 0.196 & 0.763 & 0.021\\ 0.000 & 0.031 & 0.237 & 0.732 & 0.000\\ 0.010 & 0.124 & 0.268 & 0.567 & 0.031\end{bmatrix}$$

$$R_F=\begin{bmatrix}0.082 & 0.340 & 0.505 & 0.072 & 0.000\\ 0.278 & 0.536 & 0.186 & 0.000 & 0.000\end{bmatrix}$$

Using the weighted average fuzzy operator, the fuzzy evaluation matrix was combined with the weights obtained from the analytic hierarchy process to calculate the evaluation weight vector for each criterion-level indicator as follows:

$$T_A=\omega_A\circ R_A=(0.052 \quad 0.182 \quad 0.205 \quad 0.540 \quad 0.022)$$

$$T_B=\omega_B\circ R_B=(0.715 \quad 0.172 \quad 0.113 \quad 0.000 \quad 0.000)$$

$$T_C=\omega_C\circ R_C=(0.289 \quad 0.525 \quad 0.182 \quad 0.005 \quad 0.000)$$

$$T_D=\omega_D\circ R_D=(0.336 \quad 0.467 \quad 0.169 \quad 0.028 \quad 0.000)$$

$$T_E=\omega_E\circ R_E=(0.001 \quad 0.041 \quad 0.224 \quad 0.688 \quad 0.046)$$

$$T_F=\omega_F\circ R_F=(0.131 \quad 0.389 \quad 0.426 \quad 0.054 \quad 0.000)$$

On this basis, the comprehensive evaluation vector S for the target layer of the selected existing nebulizer product was calculated as follows:

$$S=\omega_V\circ T_V=\omega_V\circ\begin{bmatrix}T_A\\ T_B\\ T_C\\ T_D\\ T_E\\ T_F\end{bmatrix}=(0.352 \quad 0.260 \quad 0.178 \quad 0.199 \quad 0.012)$$

Finally, through weighted calculation between the comprehensive evaluation vector S and the corresponding scores of the comment set levels, the score for the selected existing nebulizer product on a 100-point scale is obtained as N = 77.48.
